# The Diagnostic Accuracy of Cone-Beam Computed Tomography (CBCT) Imaging in Detecting and Measuring Dehiscence and Fenestration in Patients With Class I Malocclusion: A Surgical-Exposure-Based Validation Study

**DOI:** 10.7759/cureus.22789

**Published:** 2022-03-03

**Authors:** Hallaj I Alsino, Mohammad Y Hajeer, Issam Alkhouri, Rashad M.T. Murad

**Affiliations:** 1 Orthodontics, University of Damascus, Damascus, SYR; 2 Oral and Maxillofacial Surgery, University of Damascus, Damascus, SYR; 3 Toxins and Pharmaceutics, University of Damascus, Damascus, SYR

**Keywords:** specificity, sensitivity, diagnostic accuracy, gold standard, fenestrations, dehiscence, cone-beam computed tomography (cbct), class i malocclusions, crowded lower anterior teeth, periodontally accelerated osteogenic orthodontics (paoo)

## Abstract

Background

No study has evaluated the diagnostic accuracy of cone-beam computed tomography (CBCT) imaging in detecting bone defects in orthodontic patients with Class I malocclusions. This study aimed to evaluate the accuracy of CBCT in detecting dehiscences and fenestrations before orthodontic treatment compared to the gold standard i.e., the actual clinical detection of bone defects on surgical exposure.

Methods

A validation study was undertaken at the Department of Orthodontics, University of Damascus between 29 August 2018 and 1 November 2020. The sample included 16 patients who had Class I malocclusion with moderate crowding on the lower anterior teeth.

Results

The proportion of dehiscence diagnosed on CBCT images was approximately two-and-a-half times greater than that found on direct examination i.e., 42.7% versus 17.7%, respectively. The proportion of fenestrations was almost three times greater when diagnosed on CBCT images compared to the gold standard i.e., 39.5% versus 13.5%, respectively. The sensitivity of CBCT imaging in detecting dehiscence and fenestration was 100%. The specificity of CBCT imaging ranged from 45.5% to 86.7% and from 50% to 86.7% for dehiscence and fenestration detection, respectively. Also, the diagnostic accuracy of CBCT imaging ranged from 44% to 87.5% and from 56% to 87.5% for dehiscence and fenestration detection, respectively.

Conclusions

The proportion of dehiscence diagnosed on CBCT images was approximately two-and-a-half times greater than that found on direct examination, and the proportion of fenestrations was almost three times greater when diagnosed on CBCT images compared to the gold standard. The CBCT overestimates the dimensions of the linear measurements compared to those measured by the gold standard.

## Introduction

Dehiscence is a bone defect that is described as a decrease in the crestal bone margin that exposes the root surface [1]. Fenestrations are isolated areas in which the root is denuded of bone, and the root surface is covered only by periosteum and overlying gingiva [2]. The anatomical limit set by the labial and lingual/palatal cortical plate may be regarded as the orthodontic wall [3]. Exceeding this anatomical limit by sagittal movement and excessive force might lead to dehiscence and fenestration [3].

The occurrence of dehiscence and fenestration during orthodontic treatment depends on several factors, such as the direction of movement, the frequency and magnitude of orthodontic forces, and the volume and anatomic integrity of periodontal tissues [4]. Evangelista et al. reported that dehiscences were 35% more prevalent in patients with Class I malocclusion compared with Class II Division 1 patients before orthodontic treatment [5]. Yagci et al., in their study, reported that fenestrations had greater prevalence in the maxilla (Class I group, 18.83%; Class II group, 19.49%; Class III group, 14.06%), but more dehiscences were found in the mandible (Class I group, 24.02%; Class II group, 22.77%; Class III group, 42.64%) [6].

To avoid the formation of bone defects during orthodontic treatment, the alveolar morphology must be determined before orthodontic treatment through imaging, which shows bone topography and anatomy [5]. Dehiscence and fenestrations could not be visualized by traditional two-dimensional radiography because of the superimposition of contralateral cortical bony or dental structures [7,8]. Cone-beam computed tomography (CBCT) has been claimed to provide the means to visualize these defects three-dimensionally [9].

A study of 13 human dry skulls showed that the number of fenestrations detected by CBCT was more than three times higher than for direct examination (104 fenestrations by CBCT vs. 32 by direct measurement) and the number of dehiscences was found in CBCT imaging less than that of the direct examination (43 dehiscences by CBCT vs. 52 by direct measurement) [10]. However, the accuracy of CBCT imaging in detecting these alveolar defects when applied on dry skulls may not resemble the actual scenario on live subjects. Another study also showed that the CBCT images might overestimate the actual measurements because the number of fenestrations detected by CBCT was more than four times higher than that calculated by direct examination (31 vs. seven) and the number of dehiscences was more for CBCT than for direct examination (67 vs. 60) [11]. Their study included skeletal Class III patients with an anterior crossbite. Therefore, the generalizability of these results cannot be assured since their malocclusion sample was different from the ordinary patients that usually visit orthodontic clinics with different types of malocclusions. Therefore, a study is required to determine the accuracy of CBCT in detecting dehiscences and fenestrations in routinely referred patients. This study aimed to evaluate the accuracy of CBCT in detecting the presence of dehiscences and fenestrations before undergoing orthodontic treatment compared to the gold standard i.e., the actual clinical detection of bone defects on surgical exposure. The research hypothesis is that CBCT imaging can give an accurate diagnosis and measurement of fenestration or dehiscence similar to what is observed directly on the alveolar bone of the lower anterior teeth.

## Materials and methods

Study design and settings

A validation study was undertaken at the Department of Orthodontics, University of Damascus Dental School between 29 August 2018 and 1 November 2020 based on a cross-sectional study of patients undergoing orthodontic treatment in conjunction with a periodontally accelerated osteogenic orthodontics (PAOO) procedure whose presurgical CBCT images are collected routinely. Approval for this study was obtained from the Local Research Ethics Committee (IRB No.UDDS-485-20022018/SRC-3300) and was funded by the University of Damascus Postgraduate Research Budget (Ref No. 80004460115DEN).

Sample size calculation

The required sample size was calculated using the Minitab®19.1 software (Minitab LLC, Pennsylvania, USA) with an alpha level of 0.05, a power of 95%. The smallest difference in the dimensions of the bone defect to be detected between the CBCT and the direct method of measurement is 0.25 mm with a standard deviation of 0.25 mm from a previous study [10]. Employing paired-sample t-test, the required sample size was 16 patients.

Patients' recruitment and inclusion in the study

The patients were treated at the Orthodontic Department of the University of Damascus and the sample was collected from patients who were to be treated using fixed appliances in conjunction with a PAOO procedure. Their consent was taken to use their pre-surgical CBCT images and to allow for intra-operative direct measuring by the principal researchers (HIA). Initially, 40 patients with skeletal class I malocclusion with moderate crowded lower anterior teeth were screened for eligibility and only 20 of these patients met the inclusion criteria. Only 16 patients were randomly selected from the sampling frame and were included in the study. The project took place between August 2018 and November 2020.

The inclusion criteria were: (1) Class I skeletal malocclusion (ANB=2-4 assessed cephalometrically); (2) Moderate crowding of lower anterior teeth (i.e., 4 mm to 6 mm of a tooth size-arch length discrepancy); (3) Absence of anterior or posterior crossbites; (4) 1 mm to 3 mm of overjet and 1 mm to 4 mm of the overbite; (5) Adult healthy patients from both sexes within an age range of 18 to 28 years; (6) Patients planning to undergo PAOO surgery; (7) No previous orthodontic treatment (8) Good oral hygiene. Exclusion criteria were: (1) Medical, social, and psycho contraindications to oral surgery; (2) Cyst or tumor in the alveolar bone; (3) Previous orthodontic treatment; (4) Poor oral hygiene.

CBCT image acquisition

The CBCT images were taken of all patients before commencing any therapeutic intervention. The CBCT imaging was performed using the PaX-i3D Green Device (Vatech Co., Ltd. Gyeonggi-do, Korea), with 6mA, 95 kV, 9 seconds exposure time, and isotropic voxel size of 0.25 mm. All CBCT images were taken with their heads stabilized using ear rods that were placed in the external auditory meatus and the Frankfort plane parallel to the floor. Files were saved in Digital Imaging and Communications in Medicine (DICOM) format and the images were viewed through "EzDent-i and EzDent-plus" software (Vatech Co., Ltd. Gyeonggi-do, Korea).

Surgical procedure and the gold standard readings

**Figure 1:**
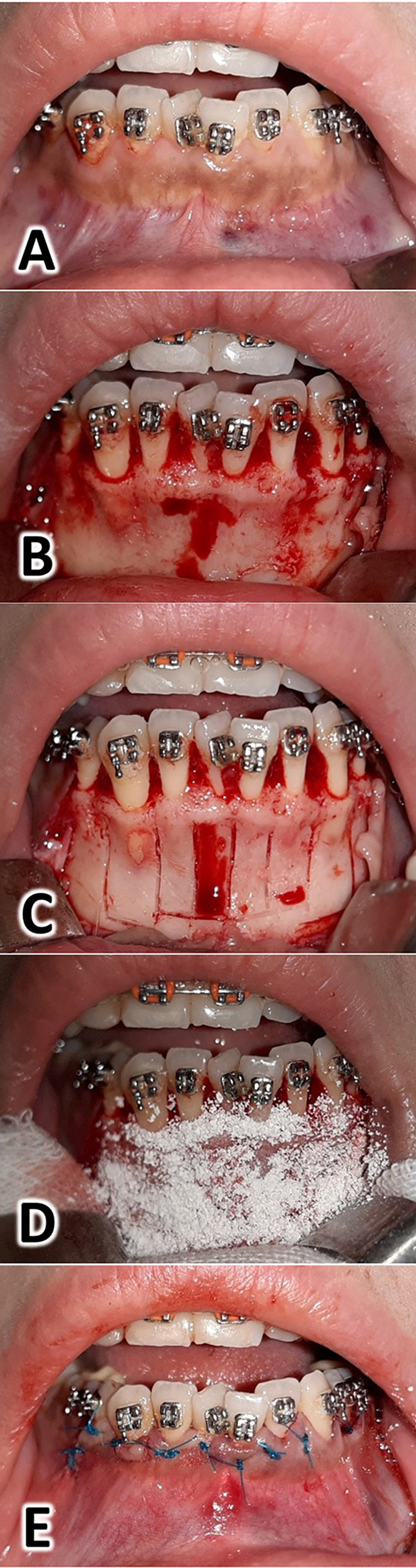
Surgical intervention procedures Patients were administered local anesthesia (lidocaine HCL 2% - epinephrine 1:80000) ahead of the surgery. A: The lower dental arch with the anterior crowding before conducting the surgical intervention, B: The full-thickness flap was elevated from the labial side only, C: The selective cortical cuts were performed between the roots of the lower anterior teeth using a piezotome, D: The xenograft using a 0.2 mm to 1.0 mm of particle size (Bone-D®, MedPark Co, Busan, Korea) was placed on the vestibular surface of the alveolar bone, E: The wound was sutured using a 2-metric nylon 3/0 suture using a reverse cutter needle (Shandong, China).

Surgery was performed at the Department of Oral and Maxillofacial Surgery, University of Damascus. A postgraduate MSc student at the Department of Oral and Maxillofacial Surgery performed all surgical interventions under the direct supervision of one of the co-authors (I.A). All surgical procedures were accomplished according to the method suggested by Bahammam [12]. Under local anesthesia, a full-thickness flap was raised labially from the distal surface of the lower right canine to the distal surface of the lower left canine. The exposed alveolar bone was washed with saline. Subsequently, the exposed alveolar bone was evaluated and the existing bone defects were recorded. A selective cortical cut was performed according to vertical cutting lines between the roots and ended with a horizontal cutting line using a piezosurgery and then the xenograft of particle size of 0.2 mm-1.0 mm (Bone-D®, MedPark Co, Busan, Korea) ) was placed, then the wound was sutured (Figure 1). Following surgery, the patients were prescribed amoxicillin 500 mg tab three times daily for one week, paracetamol 500 mg if necessary, and chlorhexidine gluconate 0.12% twice a day.

Outcome measurements

Gold Standard Measurements

**Figure 2:**
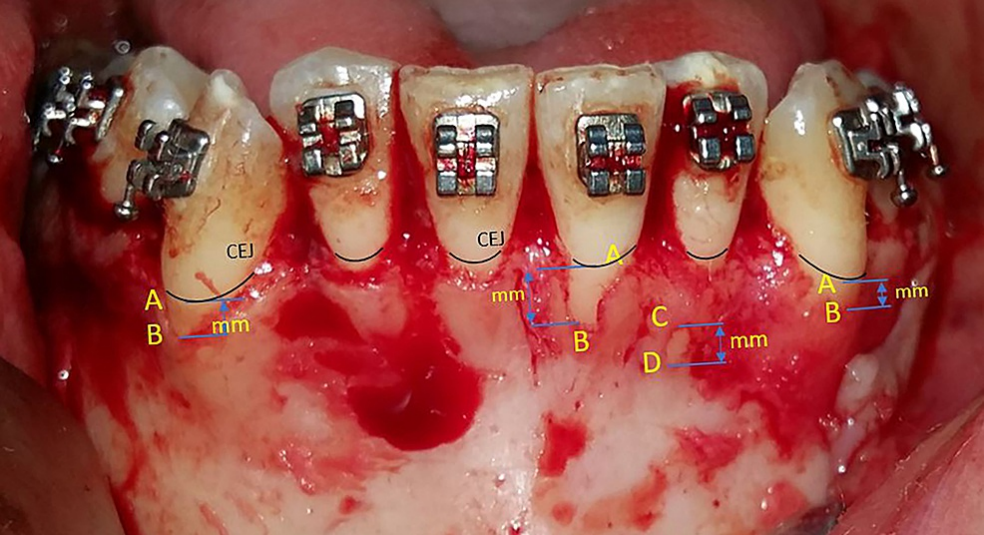
Intraoperative measurements of dehiscence and fenestration A: Cementoenamel junction (CEJ) at the labial side, B: Alveolar crest at the labial side, C: Coronal border of a fenestration, D: Apical border of a fenestration, DD: Distance between A and B measured directly in the surgical field by the calliper (mm), FD: Distance between C and D measured directly in the surgical field by the calliper (mm)

During the surgical intervention, the alveolar bone that covered the roots of the lower anterior teeth was exposed. First, the dentoalveolar bone defects diagnosed as fenestration or dehiscence were identified and counted. Then, a measurement of the height of these bony defects was carried out according to the longitudinal axis of each of the lower anterior teeth using a digital calliper. If the bone defect included the alveolar crest and the distance between the cementoenamel junction (CEJ) and the deepest point of the bone defect was greater than 2 mm (as shown in Figure 2; the distance between A and B), then it was classified as a 'dehiscence'. And if the bone defect did not include the alveolar crest and its vertical measurement was greater than 0 mm, (as shown in Figure 2; the distance between C and D), then it was classified as a 'fenestration'.

CBCT-Based Identification of Dehiscence and Fenestration and the Related Measurements

**Figure 3:**
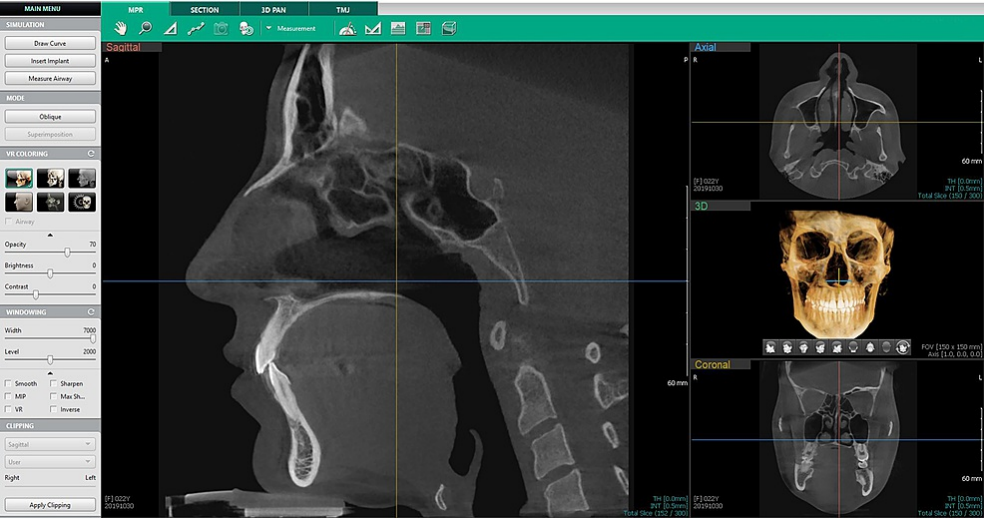
The interface of the Ez3D-i Software for viewing and manipulating images captured by the PaX-i3D Green Device (Vatech Co., Ltd. Gyeonggi-do, Korea)

**Figure 4:**
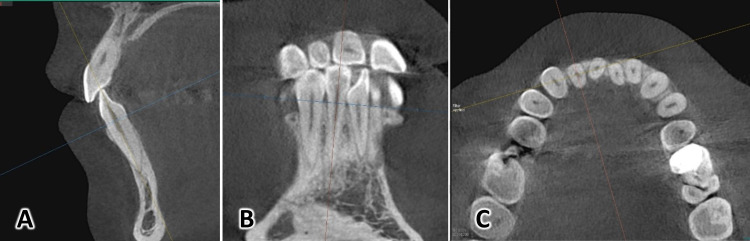
Orienting the CBCT images for the detection and measurement of fenestrations and dehiscences First, the axial plane (in blue) was moved to pass through the CEJ from the buccal and lingual sides of each tooth of the lower anterior teeth as can be seen in the sagittal (A) and coronal views (B).  Then, the coronal plane (in orange) was moved until it became parallel to the vertical axis of each examined tooth passing from the incisal edge to the apex of the root (A). Afterwards, the sagittal plane (in red) was adjusted until it passed the middle of the incisal edge and the root apex (B and C).

**Figure 5:**
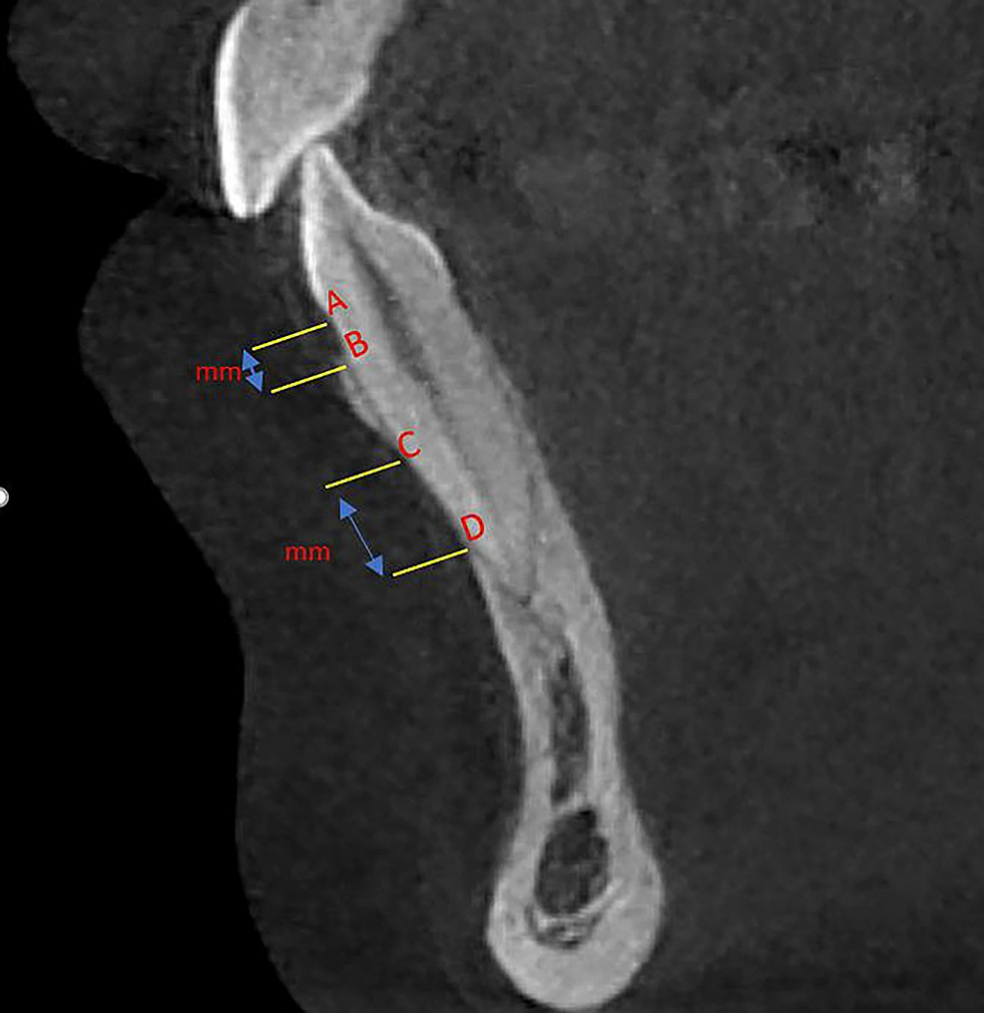
Measuring the dimensions of bone defect based on the sagittal view of the CBCT data. A: Cementoenamel junction (CEJ) at the labial side, B: Alveolar crest at the labial side, C: Coronal border of a fenestration, D: Apical border of a fenestration, DC: Distance between A and B measured by the CBCT method (mm), FC: Distance between C and D measured by the CBCT method (mm)

All CBCT images from the sagittal and coronal view were opened using EzDent-i software (Figure 3), the blue axial plane was moved to pass through the CEJ from the buccal and lingual sides of each of the lower anterior teeth. Then the orange coronal plane was moved until it was parallel to the vertical axis of the tooth to pass through the incisal margin and the apex of the root. In a third step, the red sagittal plane was moved until it reached the middle of the incisal margin and the apex of the root. Thus, the largest buccal-lingual section of the lower anterior tooth was obtained in the sagittal view, which was the appropriate section for detecting bony defects and measuring their vertical dimension (Figure 4). As a definition of an alveolar defect, any case that presented no cortical bone around the vestibular surface of the root in at least three sequential sagittal views was considered an alveolar defect. Then the dimensions of the bone defect were measured based on CBCT as in Figure 5.

Statistical analysis

Using Minitab® Version 19.1 (Minitab Inc., Pennsylvania, USA), Anderson-Darling normality tests showed an abnormal distribution of the collected data, so non-parametric tests were used. Wilcoxon signed-rank test was used to detect the difference between the two methods of measuring bone defects. Statistical significance was considered when a p-value was found less than 5%. For the multiplicity of tests, Bonferroni's correction was employed. McNemar-Bowker test was performed to detect any significant difference between the CBCT-based diagnosis of bone defects and the equivalent interpretations taken directly from the surgical field (i.e., the gold standard readings). All statistical analyses were performed using SPSS® software (version 24.0; IBM Corp., Armonk, NY, USA).

## Results

Sample characteristics

The sample included 16 patients who had skeletal class I malocclusion (ANB=3.77±1.19) with moderate crowding on the lower anterior teeth (i.e., 5.06 ± 0.87 mm of a tooth size-arch length discrepancy). The sample consisted of four males and 12 females. The age ranged from 18 to 28 years with an average of 21±3.05 (Table 1).

**Table 1:** Basic sample characteristics in terms of sex, age, cephalometric measurements, and the amount of crowding (°): in degrees; SNA: The sagittal relationship of the upper jaw to the anterior cranial base; SNB: The sagittal relationship of the lower jaw to the anterior cranial base; AND: The skeletal classification of the relationship between the upper and lower jaws; SN-SPP: The angle between the anterior cranial base (SN) and the palatal plane (SPP); SN-GoMe: The angle between the anterior cranial base (SN) and the mandibular plane (GoMe); Bjork sum: The sum of three different angles used to indicate the type of facial pattern; Jarabak ratio: the ratio of the posterior facial height to the anterior facial height; U1-SPP: The angle between the long axis of the upper incisor and the SPP plane; L1-GoMe: The angle between the long axis of the lower incisor and the mandibular plane (GoMe). L1-L1: The inter-incisal angle, SD: Standard deviation

Variable	Distribution or Mean Value (± SD)
Sex	4 males, 12 females
Age	21±3.05
SNA (°)	82.68±2.31
SNB(°)	78.73±2.44
ANB (°)	3.77±1.19
SN-SPP (°)	8.86±0.99
SN-GoMe (°)	33.94±2.57
Bjork sum (°)	392.22±2.52
Jarabak ratio	64.41±2.67
U1-SPP (°)	106.11±6.25
L1-GoMe (°)	91.26±5.40
U1-L1 (°)	135.65±6.43
Crowding (mm)	5.06 ± 0.87 mm

The proportion of dehiscence and fenestration in the collected sample

The alveolar bone overlying 96 lower anterior teeth of the mandible was examined using the direct method (intraoperative readings upon surgical exposure), the number of dehiscences was 17 with a proportion of 17.7%, whereas the number of fenestrations was 13 with a proportion of 13.5%. The greatest proportion of dehiscence was observed at the level of the lower canines (58.8%), whereas the greatest proportion of fenestration was observed at the level of the lower lateral incisor (69.2%; Table 2). In the indirect method of CBCT-based interpretation, the number of dehiscences was 41 with a proportion of 42.7%, whereas the number of fenestrations was 38 with a proportion of 39.5% (Table 2).

**Table 2:** Number and proportions of dehiscence and fenestration at the lower anterior teeth assessed directly during surgical exposure and on CBCT images 33: The left lower canine, 32: The left lower lateral incisor, 31: The left lower incisor, 41: The right lower incisor, 42: The right lower lateral incisor, 43: The right lower canine

Tooth ID	N	CBCT		Direct method	
		Dehiscence	Fenestrations	Dehiscence	Fenestrations
33	16	9(56.2%)	5(31.2%)	5(31.2%)	1(6.2%)
32	16	3(18.7%)	9(56.2%)	1(6.2%)	7(43.7%)
31	16	7(43.7%)	5(31.2%)	2(12.5%)	1(6.2%)
41	16	8(50%)	3(18.7%)	3(18.7%)	1(6.2%)
42	16	3(18.7%)	9(56.2%)	1(6.2%)	2(12.5%)
43	16	11(68.7%)	7(43.7%)	5(31.2%)	1(6.2%)
Total	96	41(42.7%)	38(39.5%)	17(17.7%)	13(13.5%)

Sensitivity, specificity, and diagnostic accuracy

There were no statistically significant differences between the CBCT and the gold standard readings in detecting the presence of dentoalveolar bone defects i.e., dehiscence and fenestration (Table 3). The sensitivity of CBCT imaging in detecting dehiscence and fenestration was 100% (Table 3). The specificity of CBCT imaging ranged from 45.5% to 86.7% and from 50% to 86.7% for the diagnosis of dehiscence and fenestration, respectively. Also, the diagnostic accuracy of CBCT imaging ranged from 44% to 87.5% and from 56% to 87.5% for the diagnosis of dehiscence and fenestration, respectively.

**Table 3:** Specificity, sensitivity, and diagnostic accuracy values of using CBCT imaging in detecting dehiscence and fenestration at the lower anterior teeth (n=16) compared to the gold standard readings †McNemar test to detect significant differences between CBCT and the gold standard readings for dehiscence and fenestration.  Bonferroni's adjustment of the alpha level was conducted (i.e., 0.05/12=0.004). * Statistically significant at p<0.004

Tooth No	Defect	Sensitivity	Specificity	Diagnostic Accuracy	P-value†
33	Dehiscence	100%	63.6%	75%	0.125
32		%100	86.7%	%87.5	0.500
31		0%	50%	44%	0.180
41		100%	61.5%	69%	0.063
42		100%	86.7%	87%	0.500
43		%100	%45.5	%62.5	0.031
33	Fenestration	%100	%73.3	%75	0.125
32		%100	57.8	%87.5	0.500
31		%100	%73.3	%75	0.125
41		%100	%86.7	%87.5	0.500
42		%100	%50	%56	0.016
43		%100	%60	%62.5	0.031

Dimensional accuracy of CBCT-based measurements of dentoalveolar bone defects

The mean differences in the measurements of vertical dehiscence dimensions in the sagittal view on CBCT versus direct intraoperative measurements (i.e., the gold standard measurements) at the level of the six lower anterior teeth ranged from 0.89 mm (the left anterior lateral incisor) to 1.61 mm (the left anterior canine; Tables 4, 5), whereas the mean differences in the measurements of vertical fenestration dimensions in the sagittal view on CBCT versus direct intraoperative measurements at the level of the six lower anterior teeth ranged from 0.55 mm (the right anterior central incisor) to 1.86 mm (the right anterior lateral incisor; Tables 6, 7).

**Table 4:** Descriptive statistics of the measurements made of dehiscence height at the lower anterior teeth (n=16 patients) using CBCT images and direct surgical exposure GS: Gold standard, SD: Standard deviation, Min: Minimum, Max: Maximum, Q1: first quartile, Q3: third quartile

Tooth No	Method	Mean height	SD	Min	Max	Q1	Median	Q3
33	CBCT	2.80	1.97	0.90	7.00	1.45	2.10	4.17
	GS	1.18	0.72	0.00	2.5	1.00	1.00	2.00
32	CBCT	1.38	0.60	0.00	2.60	0.92	1.55	1.67
	GS	0.48	0.49	0.00	1.50	0.00	0.50	0.68
31	CBCT	1.91	1.06	0.00	5.30	1.42	1.80	2.10
	GS	1.01	0.61	0.00	2.00	0.50	1.00	1.50
41	CBCT	2.36	1.88	0.00	7.70	1.52	1.95	2.30
	GS	1.07	1.39	0.00	5.00	0.12	0.50	1.37
42	CBCT	1.55	0.42	0.90	2.40	1.30	1.50	1.80
	GS	0.64	0.51	0.00	2.00	0.25	0.50	1.00
43	CBCT	2.64	1.87	0.70	8.80	1.52	2.25	3.15
	GS	1.61	1.10	0.25	5.00	1.00	1.15	2.00

**Table 5:** Inferential statistics of the measurements of dehiscence height at the lower anterior teeth (n=16 patients) using CBCT images and direct surgical exposure † Wilcoxon Signed Ranks Test, with the Bonferroni's adjustment of alpha level (i.e., 0.05/6 = 0.008), * p < 0.008 (significant difference), GS: Gold Standard, SD: Standard deviation

Tooth No	Method	Mean difference	SD	95% Confidence Interval of the Difference		P-value†
				Lower	Upper	
33	CBCT	1.61	1.71	0.70	2.53	<0.001*
	GS					
32	CBCT	0.89	0.48	0.63	1.16	<0.001*
	GS					
31	CBCT	0.90	1.26	0.23	1.58	0.007*
	GS					
41	CBCT	1.29	0.77	0.88	1.70	<0.001*
	GS					
42	CBCT	0.91	0.62	0.58	1.25	<0.001*
	GS					
43	CBCT	1.03	1.03	0.48	1.59	<0.001*
	GS					

**Table 6:** Descriptive statistics of the measurements of fenestration height at the lower anterior teeth (n=16 patients) using CBCT images and direct surgical exposure GS: Gold Standard, SD: Standard deviation, Min: Minimum, Max: Maximum, Q1: first quartile, Q3: third quartile

Tooth No	method	Mean height	SD	Min	Max	Q1	Median	Q3
33	CBCT	1.34	2.31	0.00	6.80	0.00	0.00	3.20
	GS	0.07	0.27	0.00	1.00	0.00	0.00	0.00
32	CBCT	1.62	1.64	0.00	4.50	0.00	1.55	3.27
	GS	0.76	0.93	0.00	2.25	0.00	0.00	1.87
31	CBCT	0.91	1.70	0.00	5.70	0.00	0.00	1.47
	GS	0.10	0.43	0.00	1.75	0.00	0.00	0.00
41	CBCT	0.55	1.32	0.00	4.20	0.00	0.00	0.00
	GS	0.00	0.00	0.00	0.00	0.00	0.00	0.00
42	CBCT	2.11	2.00	0.00	5.60	0.00	2.35	3.50
	GS	0.25	0.68	0.00	2.00	0.00	0.00	0.00
43	CBCT	1.38	1.76	0.00	4.30	0.00	0.00	3.27
	GS	0.17	0.68	0.00	2.75	0.00	0.00	0.00

**Table 7:** Inferential statistics of the measurements of fenestration height at the lower anterior teeth (n=16 patients) using CBCT images and direct surgical exposure † Wilcoxon Signed Ranks Test, with the Bonferroni's adjustment of alpha level (i.e., 0.05/6 = 0.008), * p < 0.008 (significant difference), GS: Gold Standard, SD: Standard deviation

Tooth No	Method	Mean height	SD	95% Confidence Interval of the Difference		P-value†
				Lower	Upper	
33	CBCT	1.27	2.25	0.07	2.47	0.063
	GS					
32	CBCT	0.86	0.88	1.33	3.89	0.004*
	GS					
31	CBCT	0.81	1.40	1.56	2.30	0.063
	GS					
41	CBCT	0.55	1.32	1.26	1.66	0.250
	GS					
42	CBCT	1.86	1.83	2.84	4.06	0.002*
	GS					
43	CBCT	1.21	1.60	2.06	3.05	0.016
	GS					

## Discussion

This is the first validation study of the diagnostic accuracy of CBCT imaging in the detection of dehiscence and fenestration of the alveolar bone overlying lower anterior teeth in patients with class I malocclusions who were referred to have their teeth aligned at a teaching hospital. A previous validation study by Sun et al. included skeletal class III patients with an anterior crossbite, whose treatment plan included orthognathic correction [11]; and the generalizability of their findings are only limited to a very specific type of malocclusion which represents normally 5.93% from the general population [13]. Therefore, the intention was to include a broader spectrum of patients with class I malocclusion who had been offered the possibility to correct their lower anterior crowding by a PAOO procedure. This study aimed to explore two different aspects of diagnostic accuracy, i.e., the accuracy of CBCT imaging in diagnosing the presence or absence of a defect (a qualitative binary outcome) as well as the dimensional accuracy of the CBCT-based measurement of the defect size (a quantitative continuous outcome).

When dehiscence and fenestration were evaluated regarding their presence using the two methods, the proportion of dehiscence diagnosed on CBCT images was approximately two-and-a-half times greater than that found on direct examination, i.e., 42.7% versus 17.7%, respectively. The proportion of fenestrations was almost three times greater when diagnosed on CBCT images compared to the gold standard, i.e., 39.5% versus 13.5%, respectively.

The results of the current study may converge with those in the study of Evangelista et al. on the assessment of the presence of bone defects using CBCT, where the percentage of dehiscence and fenestrations in the lower jaw in patients with class I before undergoing orthodontic treatment was 57.35% and 36.51%, respectively [5].

However, the results of the current study differed from the findings of Yagci et al., in patients with class I malocclusion before orthodontic treatment where the percentage of dehiscence and fenestrations in the lower jaw were 24.02% and 1.73%, respectively [6]. The difference may be attributed to the use of a relatively large voxel size of 0.3 in the later study by Yagci et al. [14]. Also, they determined that the criterion for detecting the presence of a bone defect was to appear on three sequential sagittal slides, which may have affected their findings.

The study of Sun et al., which included 14 patients with skeletal class III, had approximately close proportions of dehiscence at the lower anterior teeth level when assessed three-dimensionally and directly (63.5% and 55.5%, respectively), whereas the CBCT-based proportion of fenestrations was 5.4 times greater than that of direct assessment (25.4% and 4.7%, respectively) [11]. This may be due to the decreased thickness of the buccal bone overlying the roots of the lower anterior teeth in patients with skeletal class III compared to the standard values [15] with the resultant lack of accuracy.

In the study of Leung et al., which included thirteen human skulls, the proportion of CBCT-based detected fenestrations was 3.25 times greater than that of the gold standard readings (85.2% and 26.2%, respectively), whereas the proportion of CBCT-based diagnosed dehiscences was less than that of direct diagnosis on the skull (35.2% and 42.6%, respectively) [10]. The disagreement between the study of Leung et al. and the current findings can be attributed to the use of dry skulls which did not resemble the clinical reality with the absence of important anatomical components such as the periosteum, oral soft tissues, and skin [14]. In addition, the CBCT images were of 0.38 mm voxel size, which could have affected the diagnostic accuracy of the captured CBCT images [16].

In this study, the sensitivity to dehiscence and fenestration detection was as high as 100%, whereas the specificity ranged from 45.5% to 86.7% and from 50% to 86.7% for the dehiscence and fenestration detection, respectively. Thus, with a high sensitivity value and a moderate to high specificity value, CBCT imaging can be considered a reasonably acceptable tool for detecting dehiscence and fenestration.

When looking at the findings of Sun et al., the sensitivity for the detection of dehiscence and fenestration was 83% and 71%, respectively. In this study, the specificity for the detection of both bone defects was 73% and 77%, respectively [11]. The results of the current study differed from those of Sun et al. because the sample of patients in the latter study had a class III skeletal malocclusion with a decreased thickness of the alveolar bone overlying the mandibular anterior teeth [15] in which CBCT imaging has poor accuracy and reliability when measuring buccal alveolar thickness, particularly when the alveolar thickness is thin [17].

Leung et al. reported sensitivity and specificity values of 80% for CBCT images in the detection of dehiscence, whereas the corresponding values when detecting fenestration were less than 40% and greater than 95%, respectively. The differences between this study and our findings can be attributed to the same reasons given above about Leung et al.'s use of dry skulls [14].

The mean measurements of vertical dehiscence and fenestration using CBCT images were greater than that obtained directly from the surgical field. This indicated that CBCT imaging overestimates the actual measurements. The differences between the 3D-based measurements and the gold standard measurement were statistically significant for both bone defects. The current findings are in agreement with the results of Sun et al. [11].

The current validation study has some limitations. The investigation about CBCT diagnostic accuracy was confined to the mandibular anterior segment. Therefore, future research work should be oriented towards the other segments of the upper and lower jaws at which dehiscence and fenestration could occur. Another limitation of the current work is its dependence on only one observer in detecting bone defects using CBCT images and intraoperative direct assessment (i.e., the principal researcher), therefore, inter-observer reliability in detecting these alveolar bone defects was not evaluated. This element is very important since it has been shown in several publications that inter-observer reliability in reading and interpreting CBCT images when diagnosing orthodontic-related problems can significantly affect the diagnostic accuracy of this imaging technique [18]. In addition, the employed CBCT apparatus produced 3D data with a voxel size of 0.25 mm. Testing other systems with smaller voxel sizes would provide additional information about the possibility of enhancing the diagnostic accuracy of CBCT imaging.

## Conclusions

The CBCT-based proportion of dehiscence was approximately two-and-a-half times greater than that diagnosed on direct examination, whereas the proportion of fenestrations was almost three times greater in CBCT images than that of direct assessment. The sensitivity of CBCT images was high, whereas the specificity ranged from moderate to high. And so, CBCT imaging can be considered a reasonably acceptable tool for detecting dehiscence and fenestration in orthodontic patients. However, CBCT-based measurements of the vertical dimensions of both bone defects appeared to be overestimated compared to the actual measurements.
